# Outsourcing Trouble: A Home International Comparison of Alternative Provision Across the UK

**DOI:** 10.1080/00071005.2025.2481869

**Published:** 2025-05-28

**Authors:** Sally Power, Jemma Bridgeman, Gavin Duffy, Gillean McCluskey, Alice Tawell, Annie Taylor

**Affiliations:** School of Social Sciences, Cardiff University - WISERD, Cardiff, UK; Cardiff University - WISERD, Cardiff, UK; Social Sciences, Education and Social Work, Queen’s University Belfast, Belfast, UK; School of Education, The University of Edinburgh Moray House School of Education and Sport, Edinburgh, UK; Department of Education, University of Oxford, Oxford, UK

**Keywords:** *alternative provision, school exclusion, political economies of education, home international comparison*

## Abstract

This paper explores the complex landscape of alternative provision across the UK and its implications for school exclusion. Drawing on interviews with over 400 professionals, parents, and pupils in ten selected local authorities in England, Northern Ireland, Scotland and Wales, we find marked differences in the scale and nature of provision. These differences reflect the UK’s diverging political economies of education. England’s provision reflects its preference for quasi-market mechanisms. Scotland’s reflects a commitment to inclusive education. Wales supports public provision but bears the legacy of historic control by England, while Northern Ireland’s landscape is almost entirely publicly provided. The data suggest that the scale and diversity of alternative provision does not reduce school exclusions. England has the highest rates of exclusion and the greater number and diversity of providers. Scotland has lower rates of exclusions and fewer providers. It may even be that the availability of alternative provision creates its own demand. However, the relationship between exclusion rates and alternative provision is not straightforward, nor are its implications for educational parity. The paper concludes by arguing there is a pressing need for research on the opportunity costs of alternative provision for young people and the public sector.

## 1. Introduction

All schools face the challenge of what to do with those pupils whose behaviour makes their continued presence in the classroom undesirable or impossible. According to recent media reports (e.g., Weale, 2023), this challenge has been exacerbated since the COVID-19 pandemic, with teachers reporting increased levels of pupil defiance and verbal and physical abuse. How teachers and schools respond to this challenge will depend on the range of alternatives that are available to them. Excluding the pupil from school – either for a fixed-term or permanently – is clearly one response. However, most schools try to put in place measures which are designed to reduce the likelihood of exclusion – particularly permanent exclusion.

While many schools have internal facilities, frequently referred to as ‘inclusion’ or ‘seclusion’ units, to accommodate pupils who are expelled from the classroom (see Power and Taylor, 2020, 2022), many also look outside the school. It is this external alternative provision which we focus on here. In particular, we are interested to examine the extent to which schools and teachers in the four nations of the UK have access to different alternatives for those pupils who are either at risk of exclusion or who have already been suspended or permanently excluded.

**Figure 1.**
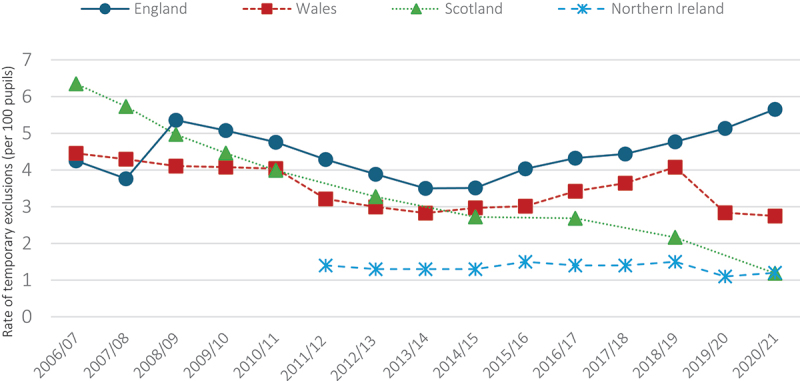
School exclusion rates (temporary/fixed term) across the UK (Tseliou *et al*., 2023)

The aim of this paper is, therefore, twofold. Firstly, exploring contextual variations in the availability and nature of alternative provision should throw light on the different political economies of education in England, Northern Ireland, Scotland, and Wales – differences which will have increased as a result of parliamentary devolution. Secondly, and relatedly, such a comparison may help us understand the different rates of school exclusion in the UK. As Figure 1 indicates, while levels of temporary (or fixed-term) exclusions are low and/or falling in Northern Ireland, Scotland and Wales, they are rising in England. There are likely to be many factors that contribute to these contrasting levels of exclusion, but it seems to us worth exploring whether the nature and availability of alternative provision might have a bearing on how schools and teachers deal with ‘troubled’ students.

Before embarking on the comparison, it is important to clarify what we mean by alternative provision, and what we are unable to consider in this paper. Our definition is as follows:

Alternative provision is planned provision where children at risk of exclusion, or who have been excluded from school, are removed from the mainstream classroom. This might be for a set period each week or full-time.

This is broader than many definitions. The term alternative provision is commonly used to refer to what Thomson and Russell (2007, p. 10) call ‘core provision’. This is institutionally based provision, such as Pupil Referral Units (PRUs) in England and Wales, and EOTAS (Education Other than at School) Centres in Northern Ireland. Pupils attend these units and centres for some, or all, of their time, often with the intention that they be reintegrated back into their original mainstream school.

We wanted to widen our definition to include interventions where young people are removed from the classroom for much shorter periods of time, even just a few hours each week. These can include a wide range of interventions that are designed to eliminate or reduce those behaviours which render the ‘troubled’ pupil at risk of exclusion, and therefore enable them to remain in the school – even if not in the mainstream classroom for all the school day or week. These are what Pennacchia and Thomson (2016) call ‘complementary programmes’.

In short, we are including in the analysis not only core provision – such as EOTAS Centres, PRUs and alternative provision academies, but also a wide range of complementary programmes for pupils who have been excluded, or are at risk of exclusion, from school. These include full- and part-time provision that may last for a few weeks or for several years. This provision may take place in a variety of settings. The provision may be commissioned by the school or the local authority (LA). It may be registered or unregistered. It may be public (funded and provided by the local authority), third sector (not-for-profit or charity) or private. The only thing we do not include in this analysis is school-provided support within the school or the kind of *ad hoc* intervention that a pupil receives if they have been required to leave the classroom because of a single incident.

Ideally, we would have liked to include within our comparison aspects of the wider landscape of education in order to gauge the overall nature of inclusivity of the education systems within the four nations of the UK. For example, we would have liked to compare the availability of placements in special schools. While special schools provide for children with a range of needs (e.g., sensory and physical, communication and cognition), not only those associated with emotional, social and wellbeing challenges, the boundary between these different categories of need is increasingly blurred. A report (Staufenberg, 2017) on the exclusion of children with special needs in England claimed that special schools are under increasing pressure to take in pupils who are ‘pushed out’ of mainstream schools. They also report that 40 new special schools are planned in England, of which 65% have autism as the primary focus. In addition to the availability of places in special schools, it is also likely that the presence of private provision may have an impact on how parents respond to the risk or incidence of their child’s exclusion from school. In addition to the private special schools, many private ‘mainstream’ schools also claim to offer enhanced opportunities for pupils with special needs, such as autism. We know that England has a far higher proportion of pupils attending private schools (6.4%) than Northern Ireland (<1%), Scotland (4%) and Wales (2%) (Green, 2022). These complexities are further compounded by jurisdictional differences in regulations governing elective home education. There is some evidence (e.g., Staufenberg, 2018) that parents will remove their children from school entirely, sometimes at the suggestion of the school, in order to avoid them being officially excluded. Unfortunately, the challenge of finding comparable data along all of these dimensions means we are unable to look at wider system-level inclusivity of the landscape. Nevertheless, we feel such comparison as we can make is important and builds on existing research.

There is already a significant amount of valuable research on alternative provision. Much of it, especially that commissioned by governments and charities, attempts to identify ‘good practice’. In England, for instance, there are various thematic reviews undertaken by Ofsted (2014, 2024). Similarly, in Wales, there are reviews (e.g., Estyn, 2015), commissioned research (McCluskey *et al*., 2013) and good practice guides (Senedd, 2019). Most of this research is based on identifying case study providers and talking to professionals and, less commonly, parents and pupils, about their experiences and perspectives. Some research focuses on the experiences of particular groups of pupils – such as girls (Dance, 2022; Russell and Thomson, 2011) and mothering schoolgirls (Vincent, 2015).

In general, the overwhelming majority of these studies focus on what we are calling core provision, such as PRUs and EOTAS Centres. This is not surprising as it is much easier to research institutions than complementary programmes and activities. There are centralised data on who attends PRUs and EOTAS centres, something which, as we shall see, is conspicuously lacking for the range of other interventions. There are a few instances of research which explore types of complementary programmes – for instance, military ethos alternative provision (Clay and Thomas, 2014) – but these studies are rare.

So, while existing research does provide useful insights into the efficacy (or otherwise) of alternative provision, it does not provide an overview of the scale and range of such provision. One notable exception is Thomson and Russell’s (2007) report for the Joseph Rowntree Foundation which attempted to map out core provision and complementary programmes in two local authorities in England. Similarly, there is very little research on the extent to which the landscape of alternative provision varies across the UK. Thomson and Pennacchia’s (2014) study for The Prince’s Trust does include case studies of providers from across the UK – examining 11 based in England, two in Northern Ireland, three in Scotland, and one in Wales. However, while their report alludes to national level variations, the small number of case studies does not enable them to draw conclusions. This paper seeks to build on this research through expanding the focus beyond case studies to provide a cross-national overview.

## 2. The Value of Home International Comparisons

This research is premised on a belief in the value of ‘home internationals’ in comparative research – a value which has increased with the diverging policy priorities that have emerged since parliamentary devolution at the end of the last century. As Raffe *et al*. (1999, p. 9) argue, for many comparative researchers, the differences between the four nations of the UK appear as something of a nuisance. However, these differences also provide rich ground for educational research. Indeed, to some extent the four nations of the UK can be seen to offer a form of ‘natural experiment’ (Leatherdale, 2019). Home international comparisons can contribute not only to greater theorisation of the relationship between the state and the education system (see, for instance, Rees, 2007) but can also provide important evidence of the consequences and efficacy of divergent policy regimes and interventions. Home international comparisons have been used to show UK-wide differences in children’s educational progress (Taylor *et al*., 2017), access to higher education (Croxford and Raffe, 2014) and teacher education (Brisard *et al*., 2007). Given that the emotional, behavioural, and wellbeing factors that contribute to pupil disaffection from school are unlikely to vary significantly across the four nations of the UK, a home internationals approach might usefully explore the policy context in which responses to such pupil disaffection are shaped.

As Bogdanor (2009) points out, the UK is a quasi-federal state which cannot be considered as either united or federated. This is particularly true in relation to education provision which has long been nationally framed – albeit to varying degrees (see West, 2023). National differences, though, have increased significantly since political devolution in 1999 which saw the establishment of the Northern Ireland Assembly, the Scottish Parliament, and the National Assembly for Wales (now Senedd Cymru). These legislatures have since developed such distinctive approaches that it is now possible to talk of four distinctive political economies of education (McCluskey *et al*., 2024).

Esping-Andersen’s (1990) classic three-fold typology of political economies of welfare predates devolution, so treats the UK as a single unit. In his typology, the UK is seen as an example of the ‘liberal’ regime, a regime in which the state favours market solutions to welfare problems. This stands in contrast to ‘conservative’ regimes, in which welfare responsibilities are seen to reside in the family, and the state only steps in when familial support is not available, and ‘social democratic’ regimes, in which the state is committed to universal provision in order to reduce social inequalities. This simple typology, though, ignores the significant and growing differences between the four nations of the UK.

There is little doubt that England continues to embody the ‘liberal’ approach, but over the last 25 years, Scotland and Wales have taken a different path. In some ways, Scotland and Wales have tried to adopt the ‘social democratic’ approach. Both countries favour a strong state and eschew market-based solutions to social welfare – albeit largely within the financial limits established by the UK Government in London. It is difficult to chart Northern Ireland’s path so clearly. Its power-sharing agreement has collapsed many times since devolution, which has effectively meant it has not had a government for extended periods of time. It is against this background that we set about trying to map out the landscape of alternative provision across the UK.

## 3. Method

Charting the similarities and differences in the landscape of alternative provision has been an iterative process that has entailed drawing data from multiple sources. As Thomson and Russell (2009) discovered in their attempt to map out provision in two local authorities in England, there are two major deficits in the data – a lack of data about programmes and a lack of data about students. While there are lists of alternative provision that are endorsed by local authorities, these are not comprehensive and do not include the many providers that schools themselves commission for individuals and for groups of pupils. We have tried to address this deficit through a systematic process of ‘trawling and mining’ (Hart, 2001) interview data from local authority officers, teachers, providers, parents, and young people to extract references to any form of alternative provision that had been used or was available.

Because of the complexity of alternative provision, we have had to derive the data from only a sample of local authorities within each country. One of the challenges in home international comparisons is trying to accommodate the different size of the four nations. Northern Ireland only has one ‘local authority’, so there was no sampling procedure. In Scotland, it was decided to sample two local authorities. In Wales, three local authorities were selected. Because of its larger size, four English local authorities were selected. In terms of population covered, it should be noted that local authorities in Wales tend to be much smaller than those elsewhere in the UK. The three in our Wales sample serve a combined population of only 700,000, compared with a population of 1,800,000 in Northern Ireland’s single education authority, 1,200,000 in the two Scottish authorities and 2,500,000 in England’s four case study authorities.

Within each of these ten local authorities, interviews with LA officers, alternative provision providers, teachers, parents, and pupils were undertaken (see Table 1 for a summary of interviews). These interviews (which include a few focus groups as well as one-to-one encounters) asked a broad range of questions about the processes of school exclusion and provision for those who have been excluded or at risk of exclusion. These interviews were then transcribed and reference to any form of alternative provision extracted. We also drew on the lists of alternative provision compiled by the local authorities themselves.

**Table 1.** Total number of interviews (includes some focus group interviews)

	*LA/agency officers*	*Alternative provision providers*	*School* staff*	*Parents/carers*	*Pupils/excludees*	*Total*
England (4 LAs)	25	13	124	9	15	186
N Ireland (1 LA)	10	4	45	4	14	77
Scotland (2 LAs)	10	5	59	7	8	89
Wales (3 LAs)	9	13	38	11	16	87
Total (10 LAs)	54	35	266	31	53	439

*Includes headteachers, pastoral leads, SEN/ALN/ASN coordinators and other school staff

Even with this ‘bottom up’ approach, it is almost certain that our inventories under-report the number of providers in a local authority. However, we believe they are sufficiently comprehensive to provide a relatively accurate picture of the landscape of provision within each authority. Moreover, the similarities between the authorities within each country provide us with some confidence that the profile of provision can be extrapolated to national level (see Power *et al*., 2024, for the profiles of alternative provision within each of the local authorities).

Once the inventories were compiled, we then coded each form of alternative provision along a number of dimensions: whether it is ‘core’ or ‘complementary’; whether it can be classified as ‘public’, ‘third sector’ or ‘private’ provision; and what kind of intervention it offers. In the following sections we begin by outlining the main differences between the four nations along these three dimensions. In providing examples of alternative provision, we have only named providers which operate across areas in order to avoid identifying the authorities, and potentially the schools, where our research was conducted. After describing the landscape of provision in each of the four countries, we then go on to discuss the significance for school exclusions, educational parity, and public accountability.

## 4. Four Contrasting Political Economies of Alternative Provision

**Figure 2.**
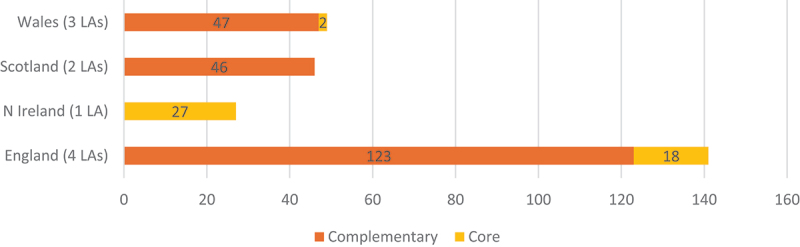
Core and complementary provision in English, Northern Irish, Scottish and Welsh authorities

There are pronounced differences in the configuration of alternative provision, and especially the relative distribution of core and complementary provision, across the four countries (Figure 2). One of the most notable differences is the lack of core alternative provision in our two Scottish case study authorities. Scotland has no designated equivalents to PRUs or EOTAS centres. Rather than sending pupils who cannot be accommodated within the mainstream classroom to separate institutions, Scottish schools have separate units (often with a focus on ‘nurture’) within the school. There is, therefore, no clearly demarcated core alternative provision in Scotland. There are, though, many complementary programmes. Scotland stands in stark contrast to Northern Ireland, which has 27 EOTAS Centres and no complementary programmes. Wales has only two core providers across the three LAs, which England has 18 across its four authorities. In the next section, we look at the differences in core provision and then in complementary provision.

### 4.1. Core Provision

One striking difference in the policy regimes in the UK is the varying extent to which the private and third sector is involved in education provision, so we were concerned to find out how far these variations are replicated in alternative provision – both core and complementary. Categorising welfare provision, including education, has become increasingly complicated. As Burchardt (2013) note, since the Conservative administration came to power in 1979, there has been successive redrawing of the boundaries between the public and the private, and, in particular, a moving away from a model of provision that is publicly provided and publicly funded. Mapping the extent of the shifting of the boundaries is complex. There is still some merit in a simple public – non-public division, not only because of clarity, but also because similar issues of governance, funding and regulation apply to non-public provision whether it is third sector or private. However, it is perhaps more useful to distinguish between non-public providers that are for-profit (private) or not-for profit (third sector) (Table 2). As Scotland has no core provision equivalent to PRUs or EOTAS centres, it does not feature in Table 2.

**Table 2.** Sector of provider of core alternative provision in English, Northern Irish and Welsh authorities

	Public		Third Sector		Private	
	n	%	n	%	n	%
England	4	19%	6	29%	11	52%
N Ireland	24	89%	3	11%	0	0%
Wales	2	100%	0	0%	0	0%

In Wales, the core alternative provision in three case study local authorities takes the form of two ‘conventional’ PRUs (of which there are a total 40 in the country as a whole). In Northern Ireland, there are 27 EOTAS Centres. Neither Wales nor Northern Ireland has any private providers of core alternative provision in the case study authorities – and there is only minimal third-sector involvement in Northern Ireland.

It is England that has the most diverse range of core alternative provision. Within our four English local authorities, only one-fifth are entirely publicly funded and publicly provided. The others are publicly funded but run by third sector organisations or, in over 50% of cases, by wholly private companies. Some of the private providers in our case study authorities run alternative provision schools throughout the country. Progress Schools (https://progress-schools.co.uk/schools/), for instance, has 13 schools located across England with fees which range from £11,000 for ‘day’ pupils to £31,500 for pupils who ‘board’. Others are standalone schools with fees for ‘day’ pupils which range from £20,000 to £45,000 per annum. There are less expensive private providers, which are often much smaller, catering for fewer than 50 pupils and charging around £10,000 per annum. Although the larger more expensive schools seem relatively well-established, the smaller private schools seem less secure. Three of the small schools identified by our interviewees as offering alternative provision have since closed, most often as a result of highly critical Ofsted inspections.

### 4.2. Complementary Programmes

There are marked differences in the scale, type, and diversity of complementary programmes in the local authorities we looked at in the four nations of the UK. Just as Scotland has no core alternative provision, Northern Ireland has no complementary programmes. Across England, Scotland, and Wales, we identified nearly 200 different providers of complementary provision in the nine authorities – but over half of these were in the four English authorities. In total, within our itineraries, England had 114 providers, Scotland had 45, and Wales had 40.

That England has a flourishing market in these programmes is illustrated not only by the number of providers, but also by the large proportion that are non-public. As Table 3 shows, wholly public providers (publicly financed and provided) are in the minority in the authorities of all three nations. Welsh authorities have the largest proportion of public providers. Only one in eight of the Scottish providers are public (as is the case with England), while the overwhelming majority are provided by charities and other not-for-profit organisations. In England, the majority of providers in the case study authorities are private companies. As Northern Ireland has no complementary provision, it does not feature in Table 3.

**Table 3.** Sector of provider of complementary programmes in English, Scottish and Welsh authorities

	Public		Third Sector		Private	
	n	%	n	%	n	%
England	14	13%	37	33%	60	54%
Scotland	6	13%	37	82%	2	4%
Wales	8	20%	22	55%	10	25%

It is not only that the presence of private, third sector and public provision varies in these three countries where there are complementary programmes, there is also an interesting variation in the *kind* of programmes that are available. Although there is some variation between the local authorities within each country (and especially between the rural and urban authorities in England), there are also marked similarities, which suggest country level difference (again, a breakdown of kinds of provision at local authority level can be found in Power *et al*., 2024).

Complementary programmes offer a wide range of activities which are designed to benefit the young person. They can be broadly grouped into six main categories: arts-based, physical exercise, nature-focused, vocational, therapeutic, and tutoring. Some programmes make claims across these categories, but they generally privilege one dimension. We briefly look at each of these types before comparing across the three nations.

*Arts-based programmes* typically provide the young person with a range of creative experiences, such as music, drama and film. For example, *Media Academy Cymru* uses ‘acting as the engagement hook … restoratively through media and creative approaches, delivering localised solutions that engage and empower individuals with the skills and self-esteem to succeed and make a positive contribution to Welsh society.’ (https://mediaacademycymru.wales/).

*Physical exercise programmes* typically emphasise the benefits of team-building and physical exertion. Local football and rugby clubs often provide programmes for disengaged pupils, but there are also UK-wide providers. *School of Hard Knocks*, for instance, is a charity which operates across England, Scotland and Wales and provides ‘a unique combination of rugby-based sessions, specialist mentoring and indoor group discussion’ (https://www.schoolofhardknocks.org.uk/). Since COVID, the organisation reports a 485% increase in participants being placed on the programme by their schools. The *Motivational Preparation College for Training* offers a range of different military activities in schools in England and Wales (https://mpct.co.uk/). *Empire Fighting Chance*, which again operates across England and Wales uses ‘the street credibility of boxing to engage young people’. Sessions are designed to be ‘both fun and impactful, incorporating a range of activities such as paid work, shadow boxing drills, punchbag workouts, circuit training, and skipping.’ (https://www.empirefightingchance.org/).

*Nature-based programmes* typically bring young people into contact with animals. *Animal Antiks* (https://www.animalantiks.co.uk/therapy/), for example, is a farm which provides young people from schools in the South East of England with ‘equine assisted therapy sessions’ through which they can ‘gain confidence and get “up close” to a horse; enjoy a series of trust-building exercises with their horse; learn “horse” language and build a lasting bond’.

*Vocational programmes* explicitly offer the young person a range of skills and are often provided on a part-time basis by further education colleges and local businesses. Courses often lead to qualifications in areas such as construction, motor mechanics, hairdressing, catering and social care.

*Therapeutic programmes* cover a range of activities, such as counselling, mentoring and mindfulness. *The Spark* (Scotland) offers ‘young people aged 12–15 a safe place in which to explore the complex emotions and issues like relationships, sexuality, stress or anxiety that come with moving into adulthood’ (https://www.thespark.org.uk/). Also in Scotland, *MCR Pathways* (https://mcrpathways.org/) runs a school-based mentoring programme which claims to help ‘young people to build confidence, broaden aspirations and explore their future pathways. In England, Scotland, and Wales, the *Place2Be* (https://www.place2be.org.uk/) offers a range of activities, such as one-to-one counselling, group work and CBT-informed therapy.

*Tutoring programmes* attempt to remedy deficits in basic skills. *Nudge Education*, which operates mainly in England, claims to be ‘leading the way in giving chronically disengaged students the opportunity to imagine a life worth living and re-engage with education’ (https://nudgeeducation.co.uk/). Their provision is endorsed by the Government’s National Tutoring Programme (NTP), which means that they can offer ‘heavily subsidised’ tuition (up to 60%) through schools’ NTP budgets. *Tute* (https://tute.com/) points to the growing need for alternative provision and the difficulty of finding placements in core provision. The private company operates across England and Wales and offers online complementary tuition for pupils out-of-classroom – ‘bridging the gap between home and school with consistent and continuous provision’.

**Figure 3.**
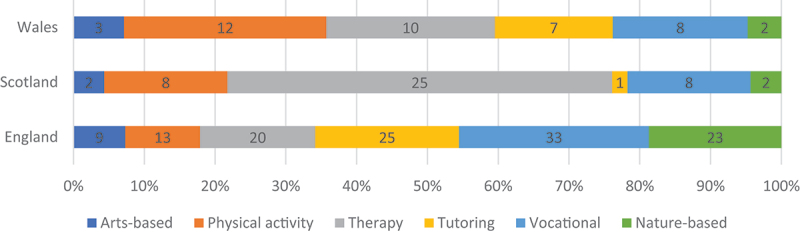
Types of complementary programme in English, Scottish and Welsh authorities

As we can see in Figure 3, the availability of these different types of complementary programmes varies across the authorities in England, Scotland, and Wales. This may reflect different national cultures, as well as different political economies. For example, the largest number of tutoring programmes can be found in England, which may reflect its performance-driven culture, which we discuss later. While Wales also has some tutoring provision, we only identified one such programme in our Scottish authorities. In contrast, these authorities appear to have a much higher proportion of therapeutic programmes offering counselling, mentoring and mindfulness. In Wales, it is physical activity that forms the focus for most complementary programmes – especially military programmes, boxing, and rugby.

In summary, this home international comparison has revealed some striking differences in the landscape of alternative provision – differences that will reflect the broader political economies of education in the UK. England’s landscape of alternative provision displays the characteristics the country’s education system as a whole and the ideological preference for quasi-market mechanisms based on diversity and choice – albeit here the choice is generally exercised by the school rather than the parent. In the four English authorities considered here, alternative provision (both core and complementary) is highly diversified, with extensive amounts of provision being offered by private providers. Of particular note is the significant number of tuition providers.

Scotland’s landscape of alternative provision appears to reflect the Scottish Government’s explicit commitment to inclusive education. There is a complete absence of core provision for pupils because such provision takes place within units operating within mainstream schools. In terms of complementary programmes, this is largely offered by the third sector rather than public or private organisations.

Wales’ landscape of alternative provision appears to bear the legacy of its historic control by England, alongside the Welsh Government’s commitment to retaining public provision. So, the presence of public providers is stronger than in England, but Wales has more of a ‘market’ of providers, with greater diversity of provision of complementary programmes and a higher number of private providers than Scotland.

Northern Ireland’s landscape of alternative provision is the most distinctive of all, with no evidence of a ‘market’. There is only core alternative provision, which is almost entirely publicly provided, with minimal third sector involvement. There is a complete absence of any complementary programmes operating outwith the core provision. The explanation for the enduring dominance of public provision in Northern Ireland can only be speculative but may be attributed to successive periods where the country has had no devolved government due to difficulties with the political power-sharing arrangements as well as a lack of priority and focus on these issues.

## 5. Discussion

### 5.1. Alternative Provision and School Exclusions

One of the reasons for undertaking this home international comparison has been to examine whether the nature and availability of alternative provision has any bearing on the rate of school exclusions across the UK. One might imagine that having a range of diverse activities designed to reengage children and young people who have difficulties in the mainstream classroom would lead to lower levels of school exclusion – indeed the potential of alternative provision to reduce exclusions is often made by the providers themselves. However, the contrasting levels of provision we have reported here, do not support this argument. If it were the case, England, with its flourishing market of alternative provision, would have the lowest levels of school exclusion. As we discussed in the Introduction, England’s rates of school exclusion continue to exceed those of Northern Ireland, Scotland, and Wales. It has been suggested that, in previous years, reductions in levels of permanent school exclusions have been achieved through higher rates of placement in alternative provision (Malcolm, 2018). However, this analysis would suggest that England has higher rates of school exclusion *and* more alternative provision than the rest of the UK.

It might be argued that the higher levels both of alternative provision and of school exclusion result from England’s policy of increasing competition between schools. Over the last 40 years, England, unlike the other three countries, has introduced a range of measures to bring quasi-markets into education, including facilitating parental choice and publishing school performance data which are compiled to make to school ‘league tables’ (Bradley, 2020; Whitty *et al*., 1998). Trotman *et al*. (2019, p. 219), on the basis of research in three English local authorities, reported increasing numbers of pupils being referred to alternative provision as a consequence of their exposure to performative school cultures. So, while there may not be a clear association between the availability of alternative provision and school exclusion rates, it is possible that without significant access to alternative provision, exclusion rates in England may be even higher than they are now.

However, there may be many other factors that contribute to the divergent rates of exclusion across the UK. For example, many schools, probably in all four countries, have some kind of unit or room where disruptive or disengaged pupils are sent for some, or all, of their time. Although there will be wide variations in the experiences these units offer, there are growing concerns about their widespread use and the quality of education that pupils might receive (e.g., Barker *et al*., 2010; Sealy *et al*., 2023). Although these kinds of practices are likely to occur all over the UK, it is probable that they are more pronounced in those contexts where official exclusions are actively discouraged. As Munn *et al*. (2000, p. 76) argue, ‘if pressure is placed on schools to reduce formal exclusion we can anticipate an increase in informal exclusion and in internal exclusions’. Where policies strongly disparage school exclusion – as they do in Scotland and Wales, schools are incentivised to find other ways of dealing with ‘difficult’ students that may also have deleterious – but hidden – consequences for students (and schools).

We also need to consider the possibility that the data on the divergent rates of exclusion are problematic. We know many pupils experience unofficial exclusion – variously described as ‘off-rolling’ and ‘informal’ exclusions. Because these practices are unlawful, there are no data on their frequency. An IPPR report (Gill *et al*., 2017) claims that, in England, five times as many children are being ‘educated’ off school registers than the official data would suggest – with many tens of thousands more being ‘lost’ from school registers illegally. But the figure may be even higher elsewhere. It is possible that levels of unofficial exclusion are higher in those contexts, particularly Scotland and Wales, where exclusion is strongly discouraged, and where high rates of exclusion can carry sanctions for schools (see Power and Taylor, 2020).

However, while the availability of diverse kinds of alternative provision may or may not be a preventative factor in school exclusion, it seems likely that, to some extent, supply will create demand. When a school is faced with the challenge of a disengaged or disruptive pupil, the availability of a wide array of alternative ‘solutions’ must be inviting. Whether alternative provision – core or complementary – does provide ‘solutions’ that have implications for inequalities is something we consider next.

### 5.2. Issues of Parity

It is clear from this analysis that there are wide variations between the four countries of the UK in terms of both core provision and complementary programmes. There is also variation between and within local authorities. The unevenness of the landscape raises issues of parity.

A number of reports have pointed to the lack of availability of alternative provision in some areas (e.g., Simms, 2022). The Taylor Report (2012, p. 6) noted the variation in the choice and quality of provision, problems with transport in rural areas and the fear of moving through ‘hostile’ urban areas, concluding that the ‘existence of good quality alternative provision in any one area is usually more a matter of luck than of any systematic planning’.

In addition to geographic unevenness, there is some research that suggests that there are inequalities in terms of who receives alternative provision. Malcolm’s (2018) comparison of placement into alternative provision (in this case, core provision) and permanent exclusions indicates that some groups are less likely to be given alternative provision placements in England. He argues that pupils in receipt of free school meals and those of White and Black Caribbean and Black Caribbean ethnicity are less likely to end up in alternative provision than they are to be excluded. There are also likely to be economic inequalities in terms of which schools and local authorities can afford to access alternative provision. It would appear that most of the complementary programmes are commissioned by schools. We know students with social, emotional and wellbeing issues are disproportionately present in schools serving disadvantaged communities. It is likely that school-level commissioning of alternative provision is likely to place significant financial burdens on these schools.

It is, though, difficult to determine whether the uneven availability and uptake of alternative provision has implications for educational inequalities as we do not have sufficient data on the benefits and costs of such provision – especially for the multitude of complementary programmes that are available. The evidence of core provision (PRUs, EOTAS centres) suggests they are not very effective at improving educational attainment (Meo and Parker, 2004; Taylor, 2012). There is, however, at least some monitoring of progress and some degree of external accountability with core provision. This is not the case for complementary programmes.

There are some case studies of complementary programmes that suggest positive outcomes (e.g., Pennacchia and Thomson, 2016), but there is relatively little systematic data on outcomes. There are claims made by providers about the difference their provision makes, but the evidence which underpins these claims is very weak. For the most part, the supporting evidence comprises narratives of individuals’ lives which have been ‘turned around’. Sometimes numerical indicators of success are given, but these rarely relate to educational outcomes and often appear unconvincing. For instance, *Media Academy Cymru* claims to ‘have diverted over 10,000 CYP (children and young people) away from the criminal justice system.’ Some providers do point to school-related benefits as reported by staff and pupils. For example, *School of Hard Knocks* claims that 90% of pupils feel more confident, and 89% more hopeful for the future. Without wishing to suggest that these outcomes do not matter, they do not necessarily suggest any progress in terms of academic attainment. In general, there is a danger that for pupils in alternative provision, ‘hard’ targets (e.g., passing GCSEs) are replaced with ‘softer’ objectives, such as increased self-esteem. While increasing self-esteem may be important for wellbeing, it does not appear to have any straightforward pay-off in terms of educational outcomes (Gorard *et al*., 2012).

There will also be opportunity costs for those pupils engaging in the various complementary programmes that take them out of the classroom even though they remain on the school register. There are very little data to know what these are – however, such research as there is suggests that whatever gains there are in terms of self-esteem, there are losses in terms of learning time. Sanders *et al*. (2016) tracked over 600 ‘vulnerable’ students from 13 to 17 in New Zealand. They found that while staying in school *was* a major factor in longer term success in keeping the young person ‘on track’, this was the case *only* when coupled with attending mainstream classes. The provision of additional educational resources outside the mainstream class did *not* lead to positive outcomes.

Because we have no idea of the relative efficacy of alternative provision, especially complementary programmes, it is difficult to form any definitive judgements about whether or not it should be more widely available, let alone what kind of provision is best, and for whom. There are more questions than answers – questions that merit further research. For example, it will be important to investigate whether Scotland’s lack of core alternative provision is really more inclusive than that of England, Scotland or Wales, especially in terms of current concerns about pupil experiences of units within schools. Other key questions that merit attention include whether therapeutic programmes are more or less useful for young people than tutoring? Does the proliferation of sports-based programmes in Wales really benefit pupils? And does Northern Ireland’s complete lack of complementary programmes mean that its young people are relatively disadvantaged compared to their counterparts in the UK? It is almost certainly the case that Northern Ireland will have less funding flowing from the public purse into third sector organisations and private companies and their provision will be more subject to external scrutiny. In general, the issue of the cost of alternative provision – in terms of opportunity costs for the young people and public sector funding in times of austerity – is currently unknown.

## 6. Conclusion

This paper has explored how the landscape of alternative provision varies in England, Northern Ireland, Scotland, and Wales. It has shown that there are striking differences in the relative availability of core and complementary alternative provision, in who provides this provision, and what form it takes. These differences reflect the diverging political economies of education of the four nations of the UK. England’s landscape of alternative provision reflects the characteristics the country’s education system as a whole and the ideological preference for quasi-market mechanisms driven by diversity and choice. It may be that Scotland’s landscape of alternative provision reflects the Scottish Government’s explicit commitment to inclusive education and high levels of third sector involvement. Wales’ landscape of alternative provision reveals the Welsh Government’s commitment to retaining public provision, but also bears the legacy of the country’s historic control by England. Northern Ireland’s landscape is the most distinctive of all, with no evidence of a ‘market’ in alternative provision. Provision here is publicly provided with minimal third sector involvement and no diversity of institutional form or type.

If we are to take official school exclusion rates at face value, it would appear that the availability of diverse types of alternative provision does not reduce school exclusions. England has the highest rates of school exclusion and, on the basis of this analysis, the highest number and greatest diversity of providers. Scotland has the lowest rates of school exclusions, and relatively low numbers of providers. It may even be the case of that the availability of alternative provision creates its own demand. However, the relationship between exclusion rates and alternative provision is not straightforward. We cannot be sure about the robustness of the official exclusion data nor the number of pupils who are placed in their school’s internal units. Indeed, we have speculated that these forms of unofficial and internal exclusions may be higher for those countries, like Scotland and Wales, where school exclusion is strongly discouraged.

One of the most important outcomes of this analysis is the striking lack of evidence available. While there are data on who attends core alternative provision, there appear to be few, if any, records of who attends the complementary programmes, for how long, and with what outcome. This is not to say that individuals have not benefited from such provision, but without independent evidence, we just do not know how representative these individual narratives of success are. Moreover, because we do not know whether and how alternative provision makes a positive difference, we cannot know how its use or absence relates to educational inequalities – at local or national levels. Although we have pointed to some of the system-wide differences in the provision of alternative education, we are no closer to understanding its efficacy – either in reducing exclusions or in providing young people with worthwhile educative experiences.

7. Data Access Statement

## Data Availability

Where ethical considerations allow, supporting data are available to bona fide researchers, subject to registration, from the UK Data Service at http://reshare.ukdataservice.ac.uk/.
